# Compliance With World Health Organization (WHO)-World Federation of Societies of Anesthesiologists (WFAS) Standards for General Anesthesia at Ibn Sina University Hospital Center, Morocco

**DOI:** 10.7759/cureus.51980

**Published:** 2024-01-09

**Authors:** Wafaa Harfaoui, Hicham Ziani, Zakaria Slaihi, Manal Arfaoui, Bouchra Armel, Hamza Elhamzaoui, Lahcen Belyamani, Mustapha Alilou, Majdouline Obtel

**Affiliations:** 1 Epidemiology and Public Health, Laboratory of Community Health, Preventive Medicine and Hygiene, Faculty of Medicine and Pharmacy, Mohammed V University, Rabat, MAR; 2 Epidemiology and Public Health, Laboratory of Biostatistics, Clinical Research and Epidemiology, Faculty of Medicine and Pharmacy, Mohammed V University, Rabat, MAR; 3 Intensive Care Unit, Hospital Ibn Sina, Rabat, MAR; 4 Mohammed VI Foundation of Health Sciences, Mohammed VI University, Rabat, MAR; 5 Royal Medical Clinic, Mohammed V Military Hospital, Rabat, MAR; 6 Faculty of Medicine and Pharmacy, Mohammed V University, Rabat, MAR

**Keywords:** post-anesthesia care, anaesthesia checklists, safety, equipments, practices, standards, anaesthesia

## Abstract

Introduction: Patient safety in anaesthesia has significantly improved over the past decades, largely due to pharmacological and technological advancements, as well as the widespread adoption of guidelines and standards recommended by international organisations. This study aimed to evaluate the practice of anaesthesia and its compliance with the international standards for safe anaesthesia practice recommended by the World Federation of Societies of Anaesthesiologists (WFSA) and the World Health Organization (WHO). This study also describes the operating room within Ibn Sina University Hospital Centre (CHUIS) of Rabat, Morocco, the referral centre, with the aim of identifying its potential and shortcomings.

Methods: This was a prospective analytical descriptive study from March 1, 2021, to July 31, 2021. All facilities within an operating room and information regarding patients undergoing general anaesthesia, whether it be emergency or scheduled procedures, administered by an anaesthetist, were included. A survey form based on the WHO-WFSA International Standards for a Safe Practice of Anesthesia was used to collect data about the anaesthesia sites. Sources of information included direct observation of anaesthesia procedures, patient records, registers, and qualified anaesthesia personnel. Manual data analysis and encoding were performed using Microsoft Word and Excel (Microsoft Corporation, Redmond, Washington, United States).

Results: All the facilities within the operating rooms of CHUIS were surveyed. In total, 250 patients were recorded, with 43.6% at Ibn Sina Hospital, 18.4% in the Children’s Hospital, 14% at the National Institute of Oncology, 12% at the Specialties, 6% at Maternity Hospital Souissi, and 6% at Orangers Maternity Hospital. The median age of patients was 50 years old with 37% of them aged 36-55 years. Overall, 67.6% of these patients were admitted for scheduled interventions. Anaesthetic risk assessment showed that 67.2% of the patients were in American Society of Anesthesiologists (ASA) class I. Pre-anaesthesia consultations were conducted in 65.6% of cases, and pre-anaesthesia visits were conducted in 89.6% of cases. Anaesthesia checklists were used in 89.6% of cases. General anaesthesia, including tracheal intubation 85.2% and facemask 7.2%, was the most common type of anaesthesia. Regarding anaesthetic agents, propofol was the most used intravenous narcotic, with fentanyl still being used in most cases 92% and rocuronium in 82% of cases. Electrocardiogram, non-invasive blood pressure, and pulse oxygen saturation (SPO2) monitoring were consistently used, while capnography was not available in 6% of cases. Crystalloid fluid resuscitation was used in 91.2% of cases, and colloid resuscitation was used in 1.2% of cases. The post-anesthesia care unit (PACU) was present in 58.8% of cases. Postoperative analgesia was administered in 80% of cases. Adverse events occurred in 58.4% of cases. Preoperative transfusion strategies were employed in 18% of cases. Patient transfers to the intensive care unit were done for 18%.

Conclusion: Despite the shortcomings of the healthcare system in Morocco, our study indicates that the anaesthesia practice at CHUIS remains highly acceptable by adhering to the highest international standards.

## Introduction

Patient safety is now a ubiquitous universal term that permeates healthcare practice in multiple ways. According to the World Health Organization (WHO), patient safety is defined as “the absence of preventable harm to a patient and reduction of risk of unnecessary harm associated with healthcare to an acceptable minimum" [1]. Patient safety is a fundamental principle in anaesthesia care worldwide. The speciality of anaesthesia is considered a leader in addressing patient safety and serves as a model for enhancing patient safety in healthcare [2].

Patient safety has evolved over the past century through a variety of safety improvements, including enhanced monitoring techniques, the development and widespread adoption of practice guidelines, and other systematic approaches to error reduction [3]. Morbidity and mortality in the developed world have decreased in recent years. This is particularly true since the publication of significant reports on critical incidents in anaesthesia care [4,5] and the establishment of anaesthesia care standards [6,7]. However, the overall outcome has not substantially changed in developing countries [8-11]. This can be attributed to various avoidable contributing factors [8,9]. In order to provide guidance and assistance in maintaining and enhancing the quality and safety of anaesthesia care worldwide, anaesthesiologists, healthcare researchers, and patient safety scientists have worked diligently in coordinated efforts over the past decades to improve patient safety in anaesthesia. For instance, the International Standards for a Safe Practice of Anaesthesia were developed on behalf of the World Federation of Societies of Anaesthesiologists (WFSA) and were first published in 1992 [12]. These standards have since evolved [13], and in 2018, the WFSA and the WHO collaboratively developed international standards for the safe practice of anaesthesia [14]. The recommended standards cover crucial areas for anaesthesia safety, including professional aspects, facilities and equipment, medications, monitoring, and anaesthesia conduct. They have been advocated as an assessment tool, enabling anaesthesia departments, institutions, or countries to evaluate their compliance and needs.

This study aims to assess compliance with the international standards recommended by WFSA and WHO for the safe practice of anaesthesia at Ibn Sina University Hospital Centre (CHUIS), Rabat, Morocco. To do this, we measured the percentage of anaesthesia that meets international safety standards in the following areas: professional aspects, anesthetic practices, facilities and equipment, medications, monitoring and the course of anaesthesia. We collected data from a representative sample of general anaesthesia performed at CHUIS over a five-month period. The results of the study will be used to identify areas for improvement and to develop strategies to improve patient safety in anesthesia.

Study setting

The CHUIS is the largest healthcare structure in Morocco, with the following metrics: functional bed capacity of 2347 beds, 328,730 consultations annually, 30,054 surgical interventions annually, and 25,379 deliveries annually. CHUIS has kept pace with the technological advancements of modern medicine. Its missions have been diverse throughout its history, including providing medical care, conducting medical research with the utmost respect for the physical and moral integrity and dignity of patients, participating in university and post-university clinical and pharmaceutical medical education, training paramedical staff, and achieving multiple public health objectives set by the state [15].

## Materials and methods

This study was conducted in the operating rooms of CHUIS (Ibn Sina Hospital, Specialties Hospital Rabat, Children's Hospital Rabat, National Institute of Oncology, Maternity Hospital Souissi, and Orangers Maternity Hospital), which serves as the primary referral centre for public education in the Rabat-Salé-Kénitra region and throughout Morocco, and routinely performs major and complex surgeries. 

This was a descriptive, cross-sectional, analytical study conducted from March 1, 2021 to July 30, 2021. In total, we recorded 250 patients. Our study focused on all patients who underwent general anaesthesia in both scheduled and emergency surgery during this period and met the selected criteria. Anesthesia data were collected using a survey form completed by anesthesiologists and nurse anaesthetists. The selection of these participants was based on their direct involvement in patient care and their sole responsibility for administering anaesthesia in the operating room. The survey form was derived from the WHO-WFSA International Standards for a Safe Practice of Anesthesia and was distributed to each establishment to gather information related to anaesthesia sites, professional aspects, facilities and equipment, medications and intravenous fluids, monitoring, the conduct of anaesthesia and suggestions from anaesthetists. Designated anaesthetists at each site collected the completed questionnaires at the end of the survey period. Data analysis and coding were performed manually using Microsoft Word and Excel (Microsoft Corporation, Redmond, Washington, United States). Of the 300 collected records, 50 were deemed unusable due to incompleteness or being lost.

Inclusion and exclusion criteria

The following were included: patients undergoing general anaesthesia in the operating room of CHUIS during the study period, the equipment present in the operating room, and the PACU, and all physicians and nurse anaesthetists working in the operating room and the PACU.

The following were excluded: patients operated on using local or loco-regional anaesthesia, patients who underwent general anaesthesia outside the study period, equipment located outside the operating room and the PACU, and incorrect or incomplete questionnaires.

## Results

Surveyed facilities level 3: referral hospitals

All the facilities with operating rooms (Ibn Sina Hospital, Specialties Hospital, Children's Hospital, National Institute of Oncology, Maternity Hospital Souissi and Orangers Maternity Hospital) were surveyed. In total, we recorded 250 patients, with 43.6% (n=109) at Ibn Sina Hospital, 18.4% (n=46) in the Children's Hospital, 14% (n=35) in the National Institute of Oncology, 12% (n=30) in the Specialties, 6% (n=15) Maternity Hospital Souissi, and 6% (n=15) Orangers Maternity Hospital.

Surgery type

All included surgeries were performed with general anaesthesia with 67.6% (n=169) of those surgeries being elective surgeries and 32.4% (n=81) being done in an emergency context. The most performed surgery was visceral (n=86; 34.4%), followed by trauma surgery (n=48; 19.2%), gynaecological-obstetric surgery (n=38; 15.2%), otolaryngology/ENT (n=24; 9.6%), and neurosurgery (n=20; 8%).

Patient characteristics

**Figure 1:**
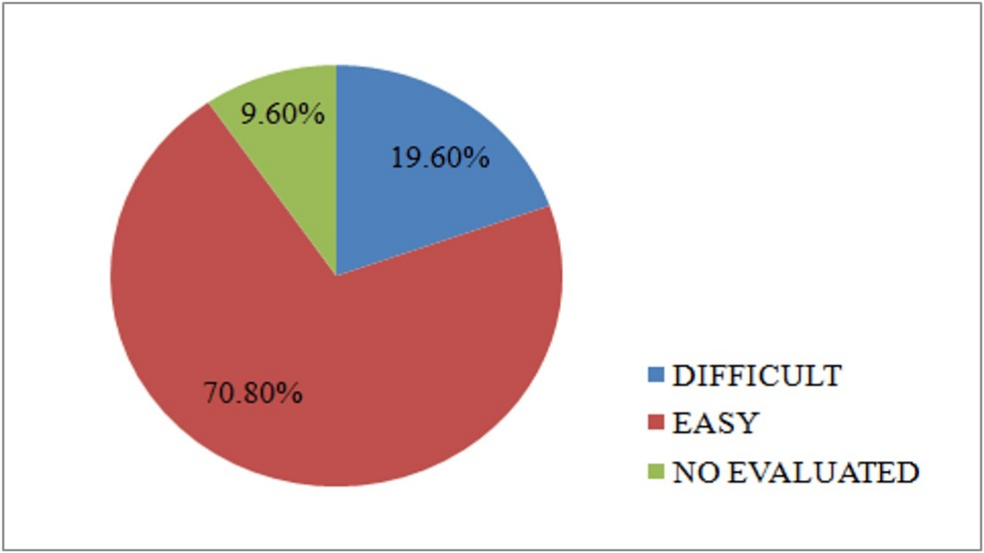
Distribution by intubation difficulty

Approximately 37.2% (n=93) of anaesthesia was performed on patients between 36-55 years old, and approximately 3.6% (n=9) were on patients over 71 years old. The distribution by gender revealed a predominance of female patients, accounting for 52% (n=130) of the cases. According to the American Society of Anesthesiologists (ASA) classification system [16], most anaesthesia cases (n=225; 90%) involved patients with minimal to mild systemic disease that is not incapacitating, classified as ASA I or II. The remaining anaesthesia was distributed among patients in ASA classes III, IV, and V. Most intubations were deemed easy (n=177; 70.8%) (Figure 1).

Professional aspects

**Figure 2:**
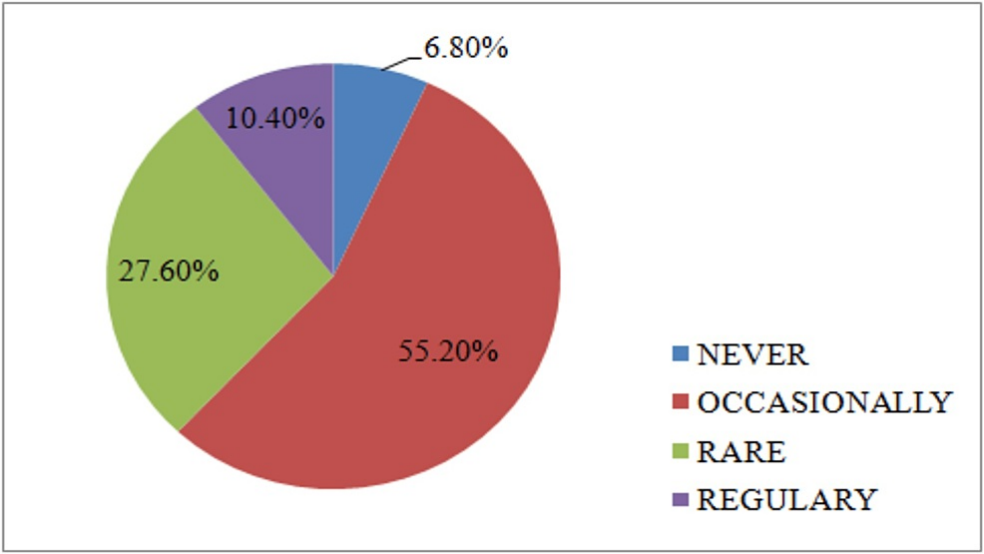
Distribution by continuous training

Anaesthesia nurses in 55.2% (n=138) of cases occasionally benefited from continuous professional training while nurses in 10.5% (n=26) of cases had regular training (Figure 2). Excellent teamwork was maintained within the healthcare staff and team (n=236; 94.4%) with only 5.6% (n=14) of staff reporting a lack of good teamwork.

Anaesthetic practices

Among all administered anaesthetics, 98% (n=245) were performed by anaesthesiologists.

Preoperative

Pre-anaesthetic consultations were conducted for 65.6% (n=164) of patients, and pre-anaesthetic visits were conducted for 89.6% (n=224). Pre-medication was administered in 73.8% (n=184) of cases and the checklist was completed in 89.6% (n=224) of cases.

Intraoperative

General anaesthesia was accompanied by tracheal intubation in 85.2% (n=213) of cases, a face mask in 7.6% (n=19) of cases and a laryngeal mask in 7.2% (n=18) of cases with various difficult intubation equipment available in each hospital (Table 1).

**Table 1:** Distribution of difficult intubation equipment

Hospitals	Difficult Intubation Equipment
Ibn Sina Hospital	Rigid stylet, Eschmann stylet, Fibroscope, Laryngeal Mask, Video laryngoscope
Children's Hospital	Straight blade, Rigid stylet, Fibroscope, Laryngeal mask, Fastrack, Video laryngoscope
National Institute of Oncology	Straight blade, Rigid stylet, Eschmann stylet, Fibroscope, Laryngeal Mask, Fastrack, Video laryngoscope, others
Specialties Hospital	Straight blade, Rigid stylet, Eschmann stylet, Fibroscope, Laryngeal mask, Fastrack, Video laryngoscope, others
Souissi Maternity	Straight blade, Rigid stylet, Eschmann stylet, Laryngeal mask, Fastrack, Cricothyrotomy, others.
Orangers Maternity	Straight blade, Rigid stylet, Eschmann stylet, Laryngeal mask, Airtraq, Video laryngoscope, others

**Figure 3:**
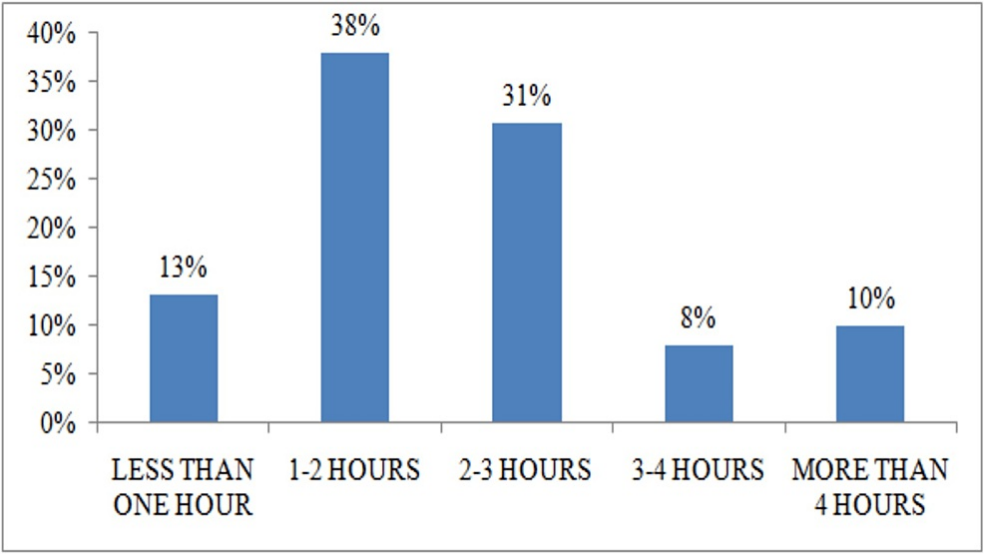
Distribution by anaesthesia duration

Approximately 13.2% (n= 33) of anaesthesia lasted less than one hour, 38% (n= 95) lasted between one and two hours, and 10% (n= 25) lasted more than four hours (Figure 3).

**Figure 4:**
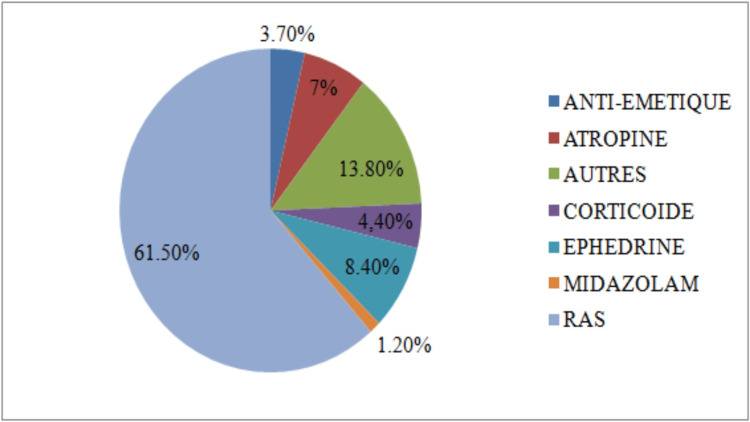
Distribution by frequently used medications

An anaesthesia sheet was filled completely in 79.6% (n=199) of cases. Propofol was the most used intravenous anaesthetic, accounting for 92% (n=230) of cases, with rocuronium being the most commonly used muscle relaxant in 82% (n=205) of cases. Fentanyl was the most commonly used opioid, accounting for 92% (n=230) of all general anaesthetics. Sevoflurane was the most used halogen in 75.2% (n=188) of general anaesthesia cases. Blood pressure monitors, ECG machines, and pulse oximeters were consistently used in all facilities. Capnography was not used in only 6% (n=15) of cases due to its absence. Crystalloid solutions were used for fluid resuscitation in 91.2% (n=228) of cases. Perioperative transfusion strategies were implemented in 18% (n=45) of cases. Intra-operative accidents occurred in 41.6% (n=104) of cases, with 26% (n=65) occurring during the operation, including cardiovascular accidents in 17.2% (n=25) of cases. Other frequently used medications included ephedrine (n=21; 8.40%), atropine (n=17; 7%), and corticosteroids (n= 11; 4.40%) (Figure 4).

Postoperative

**Figure 5:**
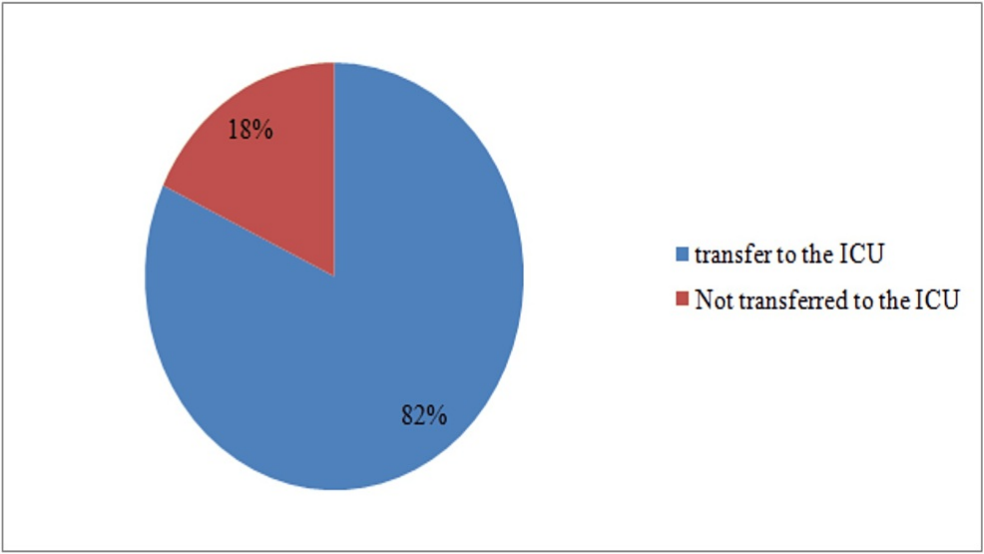
Distribution of patients by ICU transfer

Postoperative analgesia was administered in 80% (n=200) of cases, mainly using paracetamol (n=89; 35.6%), and a combination of paracetamol and nefopam chlorhydrate (n=62; 24.8%). Postoperative complications occurred in 12% (n=30) of cases, with a predominance of postoperative nausea and vomiting (PONV). A post-anesthesia care unit (PACU) was present in 58.8% (n= 147) of cases, with the most used means of monitoring then being: ECG, pulse oxygen saturation (SPO2), and non-invasive blood pressure (NIBP) monitoring. After that, 18% (n=45) of patients were transferred to an intensive care unit (Figure 5).

## Discussion

In order to assess anaesthesia practice at the CHUIS, the International Standard for a Safe Practice of Anesthesia recommended by the WHO-WFSA was used. Most interviewees met the expected and mandatory international standards for anaesthesia. These standards include elements such as the continuous presence of trained and vigilant staff, continuous monitoring of the anaesthetised patient, appropriate use of anaesthetic products, and confirmation of the implementation and monitoring of checklists. This shows that anaesthetic professionals were aware of the importance of safety and were striving to comply with international standards. However, it is important to note that international standards for anaesthesia do not cover all aspects of anaesthetic practice and may be difficult to implement in all settings; for example, the absence of a recovery room in some hospitals and the lack of use of capnography in certain cases. Despite this, to achieve the objective of improving the quality and safety of anaesthetic care, it is important to strive to achieve the highest possible standards, whether recommended or suggested. Patient safety in anaesthesia hinges on several key factors, including adequate continuing professional development, increased awareness among anaesthesia professionals, modern operating theatre infrastructure and equipment, strict safety policies and procedures, and professional motivation.

Delivering the highest quality anaesthesia remains paramount, despite existing challenges, as research has shown that improved anesthetic care is associated with a reduced risk of serious adverse events [17,18]. To ensure the highest levels of patient safety during anaesthesia, the WHO-WFSA has established three tiers of standards: highly recommended standards, which are the minimum standards expected or required, recommended standards, and suggested standards, which should be applied where resources allow [14].

Patient characteristics

The most represented age group was 36-55 years, representing 37.2% (n= 93) of our sample. In our series, women (n=130; 52%) underwent more surgical procedures than men (n=120; 48%). These observations are most likely related to the site of the study, which included two maternity hospitals. A total of 57.9% (n=144) of patients were classified ASA I, which was probably due to the young age of many patients, which also explains why 70.8% (n=177) of intubations were easy and only 19.6% (n=49) were difficult. 

Professional aspects

With regard to professional aspects, anesthesiologists were always present with a nurse anaesthetist in most surgical procedures, which is strongly recommended [14]. Only 55.20% (n=138) of nurse anaesthetists received occasional professional training. However, studies show that the continuing professional development of anaesthetists requires them to keep abreast of the latest medical knowledge and technology to provide quality patient care [19].

Good understanding existed within the team and between operating theatre staff, present in 94.40% (n=236) of cases, which is essential for success in the operating room. It can help to prevent errors, improve patient outcomes, and create a more positive working environment for everyone involved [20].

Anaesthetic practices

A total of 98% (n=245) of operations were supervised by an anesthesiologist, which may explain why preoperative visits were carried out in 89.6% of cases and preoperative consultations in 65.6% (n=164) of cases. Despite the essential role played by premedication for the physical comfort of the patient, to calm the patient's anxiety, stress, and fear, and to minimise the undesirable postoperative effects of anaesthesia and surgery [21], there is little use of preoperative premedication, which does not exceed 26.2% (n= 65) of cases.

The anaesthetic checklist is a practical support for the development and progression of patient safety in the operating theatre [22], and a tool for checking critical points. In the current study, this was carried out in 89.6% (n=224) of cases.

Tracheal intubation was used in 85.2% (n=213) of cases, while the use of a face mask was observed in 7.2% (n=18) of cases, probably because the duration of the surgical procedure was less than one hour in only 13.2% (n=33) of cases. However, the variety of difficult intubation equipment found at all the hospital sites studied is a positive finding, ensuring that clinicians have the tools they need to safely manage patients with difficult airways [23].

Anaesthesia management

With regard to anaesthesia management, the anaesthesia sheet was filled completely in 79.6% (n=199) of cases, The anesthetic record sheet is a crucial component of perioperative patient care and is a key element of the Global Patient Safety Challenge. Ensuring comprehensive and accurate anaesthesia records contributes to enhancing the safety of surgical care [24].

About the anaesthetic drugs used in this study, propofol was the most popular hypnotic agent used in the operating theatre, with a 92% (n=230) usage rate. Because it is considered to be the ideal hypnotic agent due to its unique characteristics [25], it is suggested according to the standards for intravenous drugs and fluids mentioned in the recommendations of WHO-WFSA [14]. In second place was ketamine, which is also strongly recommended. The use of rocuronium, which is suggested to induce muscle relaxation, was used in 82% (n=205) of cases. Fentanyl was the most commonly used opioid analgesic (n=30; 92%), followed by sufentanyl (n=11; 4.4%). The most commonly used halogen was sevoflurane, which is suggested, followed by isoflurane, which is recommended for use in the operating theatre. Halothane is still present in some operating theatres but is rarely used [14].

The monitoring used in all the operations included ECG, blood pressure monitoring, and pulse oximetry, while capnography was used in 94% (n=235) of cases, although capnography is another important anaesthesia monitor [26], This monitoring is strongly recommended by WHO-WFSA [14]. Using monitoring in a systematic way is essential for patient safety in the operating theatre. It enables complications to be detected early and corrective action taken if necessary. 

During surgery, crystalloids, which is highly recommended, were administered in 91.2% (n= 228) of cases, and combined with colloids in 1.2% (n= 3) of cases, which is recommended.

The most commonly used drugs in the operating room were ephedrine, atropine, corticosteroids, and antiemetics. This is because the most common adverse events that occur during surgery are cardiovascular events (n=43; 17.2%), followed by respiratory accidents (n=6; 2.4%) and PONV (n=17; 6.8%). The results show that postoperative analgesia is widely used, with 80% (n=200) of patients receiving analgesic treatment, mainly paracetamol (n=177; 35%), followed by paracetamol combined with nefopam (n= 63; 25.2%) and the combination of paracetamol and profenid (n=14; 5.6%). However, some operating theatres did not have a PACU, which is strongly recommended [14], either due to a lack of space or a lack of qualified staff. The results show that PACUs were present for 58.8% (n=147) of cases. This is an encouraging percentage, but more needs to be done to ensure that all patients receive adequate post-operative monitoring. The monitoring used in the PACU was ECG, SPO2, and NIBP in 19.6% (n=49) of cases. The rate of transfers to intensive care in 18% (n=45) of cases may be linked to blood transfusion, which was carried out in 18% (n=45) of patients.

## Conclusions

The study assessed compliance with WHO/WFSA standards for general anaesthesia in operating theatres at the CHUIS. Despite acceptable overall compliance, there are still areas requiring improvement. Our analysis revealed major shortcomings in anaesthetic practice at CHUIS, particularly in terms of continuing education for nurse anaesthetists, neuromuscular monitoring and post-anaesthetic care units. Only 55% of anaesthetists had received occasional training, which falls short of the standard strongly recommended by the WHO/WFSA. The introduction of a compulsory continuing education programme for all anaesthetic staff, particularly nurse anaesthetists, is essential to improve their skills and keep them up to date with the latest recommendations.

The total absence of neuromuscular monitoring poses a potential risk to patient safety during anaesthesia. Investment in neuromuscular monitoring equipment is recommended by the WHO/WFSA, for accurate assessment of muscle relaxation during anaesthesia. PACUs were available in only 58.8% of cases, although they are strongly recommended by the WHO/WFSA. The installation of PACUs in all operating theatres would considerably improve patient management during the critical post-operative phase. These shortcomings, if left unaddressed, could compromise patient safety. Implementing the proposed recommendations will enable CHUIS to comply with international standards and measurably improve the safety and quality of anaesthetic care for its patients.

## Appendices

Questionnaire

This questionnaire was used to collect data from theatre staff (anaesthetists and nurse anaesthetists).

For reasons of ethics and confidentiality, I would like to inform you that this questionnaire is anonymous and that the answers collected will only be used for this study and will remain confidential.

I would like to thank you in advance for your availability and your collaboration in the success of this work.

Establishment Surveyed

1. Avicenne Hospital

2. National Institute of Oncology

3. Children's Hospital

4. Speciality Hospital

5. Souissi Maternity Hospital

6. The Orangers Maternity Hospital 

The Patient

1. Sex:  M      F  , Age:   Years, Date : ../../....

2. Type of surgery:           Visceral         Gynecology          Traumatology             ENT             Urology           

                                 Neurology                     Vascular                Plastic surgery                   Thoracic                                             

3. Programmed Intervention:            Yes         No

4. Score ASA [16]:

- Normal patient:

- Patients with moderate systemic abnormality:

- Patients with severe systemic abnormality:

- Patients with severe systemic abnormality representing a constant threat to life:

- Moribund patient unlikely to survive without intervention:    

5. Premedication [21]:       Yes         No

Anaesthesist

6. Anaesthetist [14]:           Professor of Anaesthesia                 Anaesthesia Resident           Nurse Anaesthetist

7. Ongoing training [14]:     Regular             Occasionally            Rare           Never

8. A good understanding within the team [20]:       Yes      No

Anaesthetic Assessment in the Operating Theatre

9. Do you use the checklist in anaesthesia? [22]:   Yes       No

10. Preoperative Evaluation [14]:           

                        Pre-anaesthetic consultation :         Yes            No 

                        Pre-anaesthetic visit  :                  Yes               No

11. Duration of anaesthesia (minutes) :

12. Induction  (Product and Dosage) [14]:                                                                                                                     

-Narcotic:            mg

-Analgesic:          ug

-Curare:                mg

-Inhalation:          Concentration (%)

13. Airway Management:         Tracheal intubation           Face mask            Laryngeal mask

14. Intubation:             Easy                  Difficult                  Planned                Not planned

15. Difficult intubation equipment used [23]:          Yes                    No.                  If yes, which one:                                                                                                           

16. Difficult intubation tray available in your operating theatre:

17. Guedel Airway:        Yes            No.

18. Monitoring  [14]:        ECG          SpO2               NIBP               Capnography            Temperature Central venous

                                                                  pressure                              Fresh gas

19. Filling out anesthesia sheet [24] :         Yes        No

20. Post-anesthesia care unit (PACU) presence [14]:      Yes          No

                              -Monitoring used in PACU:             YES             No

                                                                             If or which :

                                 ECG          SpO2        NIBP           Capnography            Fresh gas

21. Intravenous fluid  [14]:              Crystalloid                       Colloids

22. Blood transfusion  [14]:            Yes                No

23. Postoperative analgesia (in PACU if available) [14]:            Yes          No

-Medications and doses:

-Route of administration :

-Others :

24. Other medications used during surgery:

25. Accident/Incident occurred:          Yes                      No

26. Type of incident:                     Respiratory                   Cardiovascular                      Anaphylactic reaction      

                                      Postoperative nausea vomiting                           Neurological                            Other :                     

27. Time of incident occurrence:               Preoperative             Postoperative

28. Transfer to intensive care unit:      Yes           No

29. Your expectations and suggestions for improving anaesthetic safety in your hospital:
